# Descriptive Characteristics of Undergraduate Research Associate Programs in the United States: Findings from a National Registry

**DOI:** 10.51894/001c.146562

**Published:** 2025-11-13

**Authors:** Daniel C. Keyes, Dylan Arroyo, Ghadah W. Abdulshafi, John Carzon, Margaret Beyer, Margaret Beyer, Blake Hardin, Hiba Samaha, Howard Klausner

**Affiliations:** 1 College of Osteopathic Medicine Michigan State University; 2 Department of Emergency Medicine Residency Trinity Health - Livonia; 3 School of Medicine University of Illinois Chicago; 4 Department of Emergency Medicine Trinity Health Hospital-Livonia, MI; 5 CRISP Scholar Program University of Michigan-Dearborn; 6 Department of Family Medicine Trinity Health-Livingston Hospital; 7 College of Medicine Central Michigan University; 8 Medical School University of Michigan, Ann Arbor; 9 Department of Palliative and Hospice Care Trinity Health-Livonia Hospital, MI; 10 Department of Emergency Medicine Wayne State University School of Medicine; 11 Department of Emergency Medicine Henry Ford Hospital, Detroit, MI

**Keywords:** : Research Associate Programs (RAPs), Undergraduate Research, Graduate Medical Education (GME), Clinical Research Training, Program Evaluation.

## Abstract

**Background:**

University undergraduate students often seek opportunities to gain exposure to clinical research. Physician residency training programs must engage in scholarly activities and publish their findings. “Research associate programs” (RAPs) can aid with Graduate Medical Education (GME) research. This is the first collective description of these US programs, using data from the Registry of American Research Associates Programs.

**Methods:**

The American Research Associates Program Registry (ARAPR) was started in 2014 and developed through Medline, direct familiarity, comprehensive online search, and chain-referral sampling. Data fields were selected based on a literature review and an expert panel, and included leadership, funding, research types, training, associates’ activities, university affiliation, and the selection process. Results were analyzed using descriptive statistics.

**Results:**

Responses were from 40 of 50 RAPs (80.0%) with a mean of 24 undergraduate associates (SD = 16, range 5-70) in each program. Associates worked on investigator-initiated projects (34/40, 85.0%), prospective research (35/40, 87.5%), retrospective reviews (25/40, 62.5%), and informed consent (38/40, 95.0%). Some also involved associates with data abstraction, protocol development, abstract writing, manuscript preparation, and quality improvement. Most required college course enrollment (25/40, 62.5%). Training included patient confidentiality (HIPAA) and research ethics (39/40, 97.5%).

**Conclusions:**

This survey provides the first collective descriptive insight into the structures, training, and activities of RAPs. These findings serve as a foundation for institutions considering establishing such programs and highlight the need for future research on measurable outcomes such as student trajectories, publication rates, and program impact.

## INTRODUCTION

Universities actively seek to integrate hands-on scientific research into the student experience. The American Association for the Advancement of Science (AAAS) issued a call to action for “Vision and change in undergraduate education,” which included the importance of providing opportunities to participate in “authentic research experiences”.^1^ Research associate programs offer a unique opportunity for undergraduate students to gain research experience in a clinical setting.

Postgraduate physician training programs prioritize the involvement of core faculty and trainees in clinical research. The Accreditation Council on Graduate Medical Education (ACGME) requires this by expecting faculty to “maintain an environment of research” and residents to participate in research experiences. Publications in medical journals are one way to demonstrate this involvement.^2^

Despite the ACGME requirements, there is limited guidance on strategies to promote “scholarly activity” effectively, and it is left to individual programs to define it as they see fit. The ACGME does not generally require research as a part of resident physician training. However, in recent decades, there has been increasing encouragement for GME programs to engage faculty and residents in conducting clinical research, demonstrating that clinical research is a crucial component of resident training.^2–4^ A recent survey evaluating emergency medicine (EM) residencies found that approximately 39% of EM residency programs now require original research to fulfill the scholarly activity requirement, compared to 18% fifteen years ago.^5^ The same study also identified current limitations to scholarly activity, with most programs citing study design and data collection as the principal challenges for residents.^5^ The ACGME has also emphasized quality improvement activity. This need for faculty and residents to demonstrate scholarly activity may be aided by implementing effective programs to alleviate some of those challenges.

Research Associate Programs (RAPs) can help lower barriers to research for faculty and residents, while also providing opportunities for university undergraduates. One study of residency programs identified significant barriers to residents completing scholarly activities, including inadequate time and lack of training.^6^ In the 1990s, RAPs started in New York and California to help busy physicians and residents with their research projects.^3,7^ These programs educate undergraduate, post-baccalaureate pre-medical students, and sometimes medical students to assist with study enrollment and data collection. Previous research reports have shown that RAPs can significantly enhance departmental research productivity and reduce the barriers that faculty face, while also providing pre-medical students with valuable experience and research skills.^3,7,8^ Additionally, participation in a RAP can significantly increase an individual’s chances of matriculating into medical school.^7^

The current study describes the characteristics of university- and college-based clinical research associate programs in the United States. It represents the first cross-sectional registry-based assessment of such programs, reviewing leadership structure, university affiliation, size, student type, institutional acceptance, and funding sources. Findings are intended to support the expansion or modification of existing programs and to guide the development of new initiatives by providing examples of established models. While individual RAPs have reported outcomes such as higher student acceptance into medical school and increased departmental productivity, our registry was not designed to capture outcome measures. Instead, we aimed to characterize the structures and functions of RAPs nationally, providing a descriptive baseline for future outcome-focused studies.

## METHODS

### Registry Development

The registry was explicitly designed to characterize program structures, leadership, training, and student roles, not to assess student-level or institutional outcomes. Beginning in August 2014, we developed a registry of research associate programs (RAP) through a review of existing literature and a convening of an expert panel. The complete data collected from each program is available as Supplemental Digital Appendix. Topics included RAP longevity, program size, leadership, funding, types of research, university affiliation, selection process, and other vital characteristics. Program size was evaluated by the number of students each year or term. Leadership included a physician or co-director, Ph.D. or master’s program director, research coordinator, and/or student leaders. Choices for the types of research included performance/quality improvement studies, prospective clinical research studies, retrospective medical record reviews, federally funded research, local investigator-initiated projects, industrial research, and collaborative studies with other RAPs.

### Establishing Registry

Research Associate Programs (RAPs) were initially identified through Medline, the direct familiarity of the authors, a comprehensive online search (terms *RAP, academic associate program, research associate program, undergraduate emergency medicine research)*, and chain-referral “snowball” sampling. Through chain-referral sampling, additional existing RAPs were identified by the recommendation of programs we had previously contacted. A goal of at least a 75% response rate was achieved through aggressive communication and follow-up, which enhanced the reliability of the data and decreased nonresponse bias.^9^ In the year preceding publication, the continued existence of programs was verified through their online presence and/or recontact via email or telephone. Descriptive statistical analysis was used to describe the specific characteristics of the programs.

## RESULTS

While this registry collected extensive descriptive information on program structures, leadership, and student activities, it did not capture outcome measures such as student progression, program retention, publication rates, or departmental productivity. These outcome variables were intentionally excluded from this initial registry phase but have been identified as important targets for future research.

The registry received responses from 40 of the 50 identified programs (80.0%). Twenty-seven RAPs were associated with residency programs (67.5%). A slight preponderance is based in university hospitals (25/40, 62.5%), and 15 programs (37.5%) are in community hospital GME programs. Many RAPs were in an institution with a level 1 trauma center (26/40, 65.0%). There has been a steady increase in the use of RAPs, with 14 (35.0%) being established before 2004, 9 (22.5%) between 2004 and 2009, and 16 (40.0%) being established since 2010. The respondents are primarily distributed geographically in the Eastern, Western, and Southern United States.

### Research Associate Program Operations

The majority of programs are led by a physician director (25/40, 62.5%), a physician co-director (7/40, 17.5%), and/or a research coordinator (29/40, 72.5%). There was much overlap where more than one type of leadership was provided. Many of these programs engage one or more returning associates who previously completed the program as a “chief associate” leader to help guide and organize operations. Funding sources varied considerably, with some programs hiring a specific manager primarily responsible for the program and a small percentage reporting that the program was not funded (6/40, 15.0%). The most frequently reported funding categories were the base hospital (14/40, 35.0%) or the base university (12/40, 30.0%). Many programs had more than one source of funding. Survey respondents indicated that 37.5% (15 programs) of the time, funding was partially or wholly derived from research grants.

### Research Training and Scholarly Activities

We assessed how students were trained in clinical research methods and exposure to health professions within their programs (Table 1). Almost all programs (>90%) trained their students in research ethics and institutional protocols, including Health Insurance Portability and Accountability Act (HIPAA) training, informed consent, and human subjects in research training. Most programs educated their students with some discussion of research methods (33/40, 82.5%), and nearly half of the programs (20/40, 50.0%) provided students with the opportunity to shadow clinical practitioners in the hospital setting. The majority of programs (38/40, 95.0%) required associates to obtain informed consent from human subjects for research projects, and many of them (26/40, 65.0%) were initially observed by program leadership during the consent process. Investigative types were also assessed using the questionnaire. Most of these programs carried out prospective and retrospective research projects (35/40, 87.5% and 25/40, 62.5%, respectively), and many involved students in institutional performance improvement/quality improvement projects (28/40, 70.0%). Several programs had students prepare manuscripts and/or abstracts for publication (14/40, 35.0% and 18/40, 45.0%), but only a few had students create new research projects independently (8/40, 20.0%). Table 2 shows the type of activities research associates perform for the programs included in the study.

**Table 1.** Program Training Topics

Training Topics	Percentage (% out of 40)
HIPAA Training	97.5
Human Subjects Training (e.g., CITI, NIH, etc.)	97.5
Informed Consent	95.0
Training on Active Institutional Protocols	92.5
Understanding Clinical Research Methods	82.5
Observing other students “in action”	80.0
Associates Are Observed While Practicing Consents	65.0
Practitioner Clinical Shadowing	50.0
Students Create Their Own Research Project	20.0

**Table 2.** Activities Performed by the Research Associates in the Program.

Program Activities	Percentage (% out of 40)
Consenting Patients for Research	95.0
Prospective Research	87.5
Abstract Data from Medical Records	85.0
Local Investigator-Initiated Projects	85.0
Performance Improvement/Quality Improvement	65.0
Quality Review of Consents and Data Gathered	65.0
Retrospective Chart Reviews	62.5
Abstract Preparation	45.0
Protocol Writing and/or Development	37.5
Manuscript Preparation	35.0
Federally funded research, single-site	45.0
Federally funded research, multicenter	45.0
Industrial Research (pharmaceutical, medical devices, FDA, etc.)	37.5
Collaborate with other Research Associate Programs regionally or nationally	30.0

## DISCUSSION

In this registry analysis of research associate programs in the United States and Canada, we describe their longevity, structure and organization, training modalities, and the environments in which they are found. These programs have grown since the first one was established in 1992. Almost all of these RAPs train associates in research ethics, human subjects education, HIPAA, informed consent, and how to conduct clinical research. Most programs engage their associates in prospective studies, retrospective research studies, and quality improvement projects. Some programs also allow select participants to present abstracts and co-author manuscripts. Although our registry did not capture outcome data, prior studies of individual RAPs have demonstrated substantial benefits, including increased departmental research productivity and higher acceptance rates into graduate health professions programs.

Additionally, almost all RAPs allow students to gain clinical experience by shadowing healthcare practitioners in their day-to-day operations. This shadowing opportunity may be especially beneficial for first-generation university attendees and/or disadvantaged students who often have limited exposure to role models serving in healthcare.^10,11^ The findings of this study provide a framework for future and current RAPs seeking to improve their program.

Several previous studies have described the characteristics and outcomes of individual RAPs.^7,12–14^ To the authors’ knowledge, this is the first study to describe the aggregate characteristics of RAPs in the United States and Canada. Individual RAPs have recently been reported to increase a department’s academic productivity.^14,15^ The authors report significant increases in peer-reviewed and abstract publications. Additionally, Hoonpongsimanont et al. report that their program has helped gain grant funding for research projects via pilot studies. A recent study done by Liu et al. shows that students participating in their RAP are consistently accepted to graduate health professions programs (M.D., D.O.) and other graduate school programs (Masters, Ph.D.) at significantly higher rates than the national average.^14^ The authors also report that students’ experiences within the program are often included in their applications and interviews for graduate school programs and/or jobs, thus indicating the impact of such programs on students’ careers.^8^

It is interesting to note that most programs do not incorporate the development of projects based on the student’s original research ideas. It may be that such mentorship of novices is considered too time-consuming. Abstract and manuscript preparation were not present in most RAPs, perhaps for similar reasons.

## LIMITATIONS

This study should be taken in the context of its strengths and weaknesses. The method used to establish the registry was limited to online web searches, the authors’ knowledge, and referrals from other programs. Although we suspect this registry includes a large, representative sample, some RAPs may have been overlooked. Another limitation is that some RAPs have unique characteristics that only partially fit the limited choices for each survey question, so a few program leaders wrote qualitative responses to specific questions. The study’s strengths include the high response rate of 80.0%. Studies with a high response rate are less likely to misrepresent the overall experience of the greater study population.^15^ Response rates of at least 70% to 80% have been recommended to actively mitigate non-response bias, thereby enhancing reliability.^16^

Our registry was intentionally limited to descriptive characteristics and therefore does not assess program-level or student-level outcomes. Future work should incorporate outcome measures such as student career trajectories, publication productivity, and institutional impact.

## CONCLUSION

This study provides the first national registry-based description of RAPs. While our data do not address outcomes, the descriptive framework offers a resource for program development and highlights critical areas where outcome research is needed, including student career trajectories, institutional scholarly productivity, and program sustainability.

### Ethical approval

The Saint Joseph Mercy Health System Institutional Review Board (IRB) reviewed the study and determined it to be exempt under the 2019 revision of the Common Rule (Reference Number NHSR-19-798, May 7, 2019). The return of the completed survey implied informed consent.

### Code availability

Not applicable

### Author’s contributions

The authors DK, DA, MB, BH, and HS made substantial contributions to the conception of the work. The authors DK, DA, GA, JC, MB, BH, HS, and HK contributed substantially to the data’s analysis and interpretation. All authors revised the work critically, approved the version to be published, and agreed to be accountable for all aspects of the work. All team members have read and approved the final version of the manuscript and made sure to review all aspects regarding the accuracy or integrity of the paper.

## Data Availability

Not applicable
